# Renal markers and risks of all cause and cardiovascular mortality from the Taichung community based cohort study

**DOI:** 10.1038/s41598-021-93627-5

**Published:** 2021-07-08

**Authors:** Cheng-Chieh Lin, Ting-Yu Chen, Chia-Ing Li, Chiu-Shong Liu, Chih-Hsueh Lin, Mu-Cyun Wang, Shing-Yu Yang, Tsai-Chung Li

**Affiliations:** 1 School of Medicine, College of Medicine, China Medical University, Taichung, Taiwan; 2 Department of Family Medicine, China Medical University Hospital, Taichung, Taiwan; 3 Department of Medical Research, China Medical University Hospital, Taichung, Taiwan; 4 Department of Public Health, College of Public Health, China Medical University, No. 100, Sec. 1, Jingmao Rd., Beitun Dist., Taichung, 406040 Taiwan, ROC; 5 Department of Healthcare Administration, College of Medical and Health Science, Asia University, Taichung, Taiwan

**Keywords:** Biomarkers, Risk factors

## Abstract

This study aimed to explore the associations between renal-related and arterial stiffness biomarkers with all-cause and expanded cardiovascular disease (CVD) mortality in a general Taiwanese population. This prospective community-based cohort study included 4883 subjects aged ≥ 20 years who were followed up until December 31, 2016. Renal-related biomarkers consisted of blood urea nitrogen (BUN), estimated glomerular filtration rate (eGFR), and urine albumin-to-creatinine ratio (UACR). Arterial stiffness biomarker consisted of brachial-ankle pulse wave velocity (baPWV). The death status of the subjects was ascertained by matching information from death records with the identification number and date of birth of the subjects. Cox proportional hazard models with restricted cubic splines estimated the hazard ratios and 95% confidence intervals for all-cause mortality and expanded CVD mortality. During a mean 8.3 years of follow up, 456 deaths were recorded, 146 of which were due to expanded CVD mortality. The multivariable-adjusted hazard ratios of all-cause mortality was 1.53 (95% CI 1.21–1.94) for BUN (≥ 20 mg/dL vs. < 20 mg/dL), 1.57 (1.15–2.14) for eGFR (< 90 mL/min/1.73 m^2^ vs. ≥ 90 mL/min/1.73 m^2^), 1.55 (1.25–1.92) for UACR (≥ 30 mg/g vs. < 30 mg/g), and 1.75 (1.14–2.67) for baPWV (≥ 1400 cm/s vs. < 1400 cm/s). The expanded CVD mortality was 1.89 (95% CI 1.30–2.73) for BUN (≥ 20 mg/dL vs. < 20 mg/dL), 2.28 (1.13–4.57) for eGFR (< 90 mL/min/1.73 m^2^ vs. ≥ 90 mL/min/1.73 m^2^), 2.13 (1.52–2.99) for UACR (≥ 25 mg/g vs. < 25 mg/g), and 15.73 (2.14–115.61) for baPWV (≥ 1400 cm/s vs. < 1400 cm/s). High levels of BUN, UACR, and baPWV and low levels of eGFR showed high risks with all-cause and expanded CVD mortality. Our study provides insights into screening tests to target populations at high risk of premature death due to CVD.

## Introduction

Cardiovascular diseases (CVDs) are a group of disorders of the heart and blood vessels that includes coronary heart disease, stroke, and peripheral arterial disease. CVDs are the leading cause of death worldwide^1,2^. Hypertension, hyperlipidemia, and hyperglycemia are commonly known as the major modifiable risk factors for CVDs. They promote the development of atherosclerotic arteries in the heart, brain, and extremities and may also cause hardening and rupture of blood vessels that lead to poor blood circulation. Moreover, these risk factors not only increase the chance of causing coronary heart disease but also lead to various serious complications.

Renal function has been recently treated as a biomarker of and a risk factor for the development of CVDs^3,4^. Chronic kidney disease (CKD) is reportedly an important risk factor for an increased risk for cardiovascular events and mortality, and this increase is linked to the severity of renal-related diseases^5^. Increased cardiovascular events and atherosclerosis have been widely reported in patients with end-stage CKD^6^.

Most studies that explored the relationship between renal-related biomarkers and mortality^7–15^ had been conducted in special populations, such as persons with diabetes^2^, persons with CKD^7,9^, persons undergoing percutaneous intervention^8^, persons with hypertension^12^, or persons participating in comprehensive health screening programs^15^. By contrast, studies that focused on general populations are few^10,11,13,14,16,17^. These studies evaluated the associations of urine albumin-to-creatinine ratio (UACR)^14^, estimated glomerular filtration rate (eGFR)^10,11,16,17^, or both^13^ with mortality with or without adjustment for metabolic syndrome-related markers. The results of investigations on renal-related biomarkers with mortality are inconsistent. Previous studies reported that high levels of blood urea nitrogen (BUN) are associated with an increase in all-cause mortality^7–9^. Low levels of eGFR reportedly have a positive^10,12^ or a J-shaped association^11^ or no association at all^9,13^ with all-cause or CVD mortality. By comparison, high levels of UACR reportedly have a positive association^13,14^ or no association at all^15^ with all-cause or CVD mortality. However, none of these studies that explored the association of renal-related markers with mortality considered arterial stiffness.

No prior study reported the long-term impact (more than a 10-year follow-up period) of renal-related biomarkers on the risks of mortality. Furthermore, some of prior studies exploring associations between BUN or baPWV and morality had sample sizes less than 600^7,8,18^, some studies had sample sizes between 1500–4000^9,19–22^, and only one study had a sample size over 4500. When exploring the relationship between renal markers and mortality, no prior study attempted to rule out the effects of biomarkers of metabolic syndrome and arterial stiffness simultaneously. Arterial stiffness and impaired renal function are associated with increased risk of cardiovascular events, and death^23,24^. It has been reported that endothelial dysfunction contributes to renal function^25^ and endothelial dysfunction is one of the pathophysiologic mechanisms that may affect the arterial stiffness^26^. Thus, there is a need to consider arterial stiffness when the associations between renal markers and mortality are evaluated. This study aimed to explore the associations between renal-related biomarkers and all-cause and expanded CVD mortality among 4883 participants of Taichung Community Health Study (TCHS) and Taichung Community Health Study-Elderly (TCHS-E), as well as their family members who participated in Taichung Community Health Study-family cohort (TCHS-FC). The association was investigated by adjusting for biomarkers of metabolic syndrome and arterial stiffness.

## Results

A total of 4883 participants were included in the analysis, 51.85% of which were women. The average age at baseline was 56.13 years. During the 40,320.34 person-years of follow-up period with a mean (median) follow up of 8.3 (7.2) years, 456 cases of death (9.3%) were recorded with an incidence density of 11.31/1000 person-years. Among these cases, 146 died due to expanded CVD (3.0%) with an incidence density of 3.62/1000 person-years.

Baseline characteristics of sociodemographic factors, lifestyle behaviors, health status (medical history), and cardiovascular-related factors of the subjects according to survival status are summarized in Table 1. Persons who died during the follow-up period were significantly different in terms of age, gender, BMI, educational level, marital status, smoking habits, alcohol consumption, hours spent sleeping, hours spent sitting, prevalence of heart disease, cerebrovascular disease, hypertension, diabetes, gout, cancer, WC, WHR, WHtR, SBP, DBP, PP, MAP, HDL-C, LDL-C, total cholesterol, FPG, WBC, BUN, eGFR, UACR, baPWV, pulse, SGOT, and uric acid (all p-values < 0.05).

**Table 1:** Baseline characteristics of sociodemographic factors according to survival status during 8.3 years of follow-up.

Characteristic		Overall (N = 4883)		Alive (N = 4427)	Dead (N = 456)	p-value
Age (years)		56.13 ± 15.75		54.40 ± 15.12	72.84 ± 11.36	< 0.001
**Age group (years) (no. [%])**						< 0.001
≤ 65		3308 (67.75)		3208 (72.46)	100 (21.93)	
> 65		1575 (32.25)		1219 (27.54)	356 (78.07)	
**Gender**						< 0.001
Men		2351 (48.15)		2035 (45.97)	316 (69.30)	
Women		2532 (51.85)		2392 (54.03)	140 (30.70)	
**Education attainment level**						< 0.001
Below elementary school		1027 (21.03)		845 (19.09)	182 (39.91)	
Junior high school		509 (10.42)		456 (10.30)	53 (11.62)	
High school		1235 (25.29)		1146 (25.89)	89 (19.52)	
Junior college		775 (15.87)		710 (16.04)	65 (14.25)	
Beyond University		1337 (27.38)		1270 (28.69)	67 (14.69)	
**Marital status**						< 0.001
Single		642 (13.15)		631 (14.25)	11 (2.41)	
Married		3548 (72.66)		3222 (72.78)	326 (71.49)	
Widowed		459 (9.40)		352 (7.95)	107 (23.46)	
Divorced		210 (4.30)		200 (4.52)	10 (2.19)	
Separated		24 (0.49)		22 (0.50)	2 (0.44)	
**Smoking habits**						< 0.001
Never		3720 (76.18)		3439 (77.68)	281 (61.62)	
Past		477 (9.77)		382 (8.63)	95 (20.83)	
Current		686 (14.05)		606 (13.69)	80 (17.54)	
**Alcohol consumption**						< 0.001
Never		3760 (77.00)		3445 (77.82)	315 (69.08)	
Past		201 (4.12)		155 (3.50)	46 (10.09)	
Current		922 (18.88)		827 (18.68)	95 (20.83)	
Sleep hours (hour/day)		7.06 ± 1.37		7.01 ± 1.29	7.50 ± 1.97	< 0.001
Sitting hours (hour/day)		7.71 ± 3.82		7.78 ± 3.84	7.00 ± 3.63	< 0.001
Leisure activity (MET-hour/week)^a^		315.53 ± 2524.25		293.24 ± 2609.98	531.75 ± 1433.92	0.243
**Leisure activity group (No. [%])**^a^						0.126
No regular activity		1756 (36.15)		1607 (36.49)	149 (32.82)	
Regular activity		3102 (63.85)		2797 (63.51)	305 (67.18)	
**Heart disease**						< 0.001
Yes		647 (13.25)		501 (11.32)	146 (32.02)	
No		4236 (86.75)		3926 (88.68)	310 (67.98)	
**Cerebrovascular disease**						< 0.001
Yes		152 (3.11)		96 (2.17)	56 (12.28)	
No		4731 (96.89)		4331 (97.83)	400 (87.72)	
**Hypertension**^a^						< 0.001
Yes		1380 (28.31)		1153 (26.09)	227 (49.89)	
No		3495 (71.69)		3267 (73.91)	228 (50.11)	
**Hyperlipidemia**^a^						0.172
Yes		1019 (20.96)		936 (21.24)	83 (18.28)	
No		3842 (79.04)		3471 (78.76)	371 (81.72)	
**Diabetes**^a^						< 0.001
Yes		481 (9.86)		377 (8.52)	104 (22.86)	
No		4398 (90.14)		4047 (91.48)	351 (77.14)	
**Gout**^a^						< 0.001
Yes		409 (8.38)		335 (7.57)	74 (16.23)	
No		4472 (91.62)		4090 (92.43)	382 (83.77)	
**Cancer**						< 0.001
Yes		160 (3.28)		126 (2.85)	34 (7.46)	
No		4723 (96.72)		4301 (97.15)	422 (92.54)	
**Metabolic syndrome-related markers**						
**Obesity indicators**						
BMI (kg/m^2^)		24.15 ± 3.59		24.17 ± 3.58	24.01 ± 3.65	0.279
WC (cm)		81.81 ± 10.32		81.51 ± 10.28	84.73 ± 10.28	< 0.001
WHR		0.85 ± 0.07		0.85 ± 0.07	0.88 ± 0.07	< 0.001
WHtR		0.51 ± 0.06		0.51 ± 0.06	0.53 ± 0.06	< 0.001
**Blood pressure indicators**						
SBP (mmHg)		130.65 ± 20.86		129.21 ± 20.09	144.65 ± 22.93	< 0.001
DBP (mmHg)		77.81 ± 11.67		77.56 ± 11.48	80.15 ± 13.20	< 0.001
PP (mmHg)		52.85 ± 14.90		51.65 ± 14.13	64.50 ± 17.01	< 0.001
MAP (mmHg)		95.42 ± 13.66		94.78 ± 13.34	101.65 ± 15.07	< 0.001
**Blood lipids indicators**						
TG (mg/dL)		117.42 ± 86.56		116.80 ± 85.10	123.46 ± 99.58	0.149
**HDL-C (mg/dL)**						
Men		42.68 ± 11.70		42.38 ± 11.09	44.65 ± 14.92	0.003
Women		52.33 ± 13.58		52.50 ± 13.55	49.41 ± 13.85	0.019
LDL-C (mg/dL)		121.06 ± 32.88		121.63 ± 32.92	115.48 ± 32.03	< 0.001
Total cholesterol (mg/dL)		197.36 ± 37.07		197.88 ± 36.99	192.32 ± 37.50	0.002
**Blood sugar indicator**						
FPG (mg/dL)		102.82 ± 25.97		101.76 ± 24.15	113.17 ± 38.04	< 0.001
**Inflammation indicator**						
WBC (10^3^/ul)		6.00 ± 1.66		5.97 ± 1.64	6.30 ± 1.85	< 0.001
**Renal-related biomarkers**						
BUN (mg/dL)		12.87 ± 5.04		12.45 ± 4.27	16.89 ± 8.80	< 0.001
eGFR (mL/min/1.73 m^2^)		89.13 ± 20.88		91.39 ± 19.48	67.27 ± 21.33	< 0.001
UACR (mg/g)		35.61 ± 211.54		24.15 ± 120.86	146.87 ± 569.52	< 0.001
**Arterial stiffness markers**						
baPWV (cm/s)		1.1656.30 ± 506.12		1602.67 ± 455.28	2176.92 ± 657.65	< 0.001
ABI		13 ± 0.10		1.13 ± 0.10	1.12 ± 0.11	0.135
**Others makers**						
SGPT (IU/L)		25.87 ± 21.47		25.96 ± 21.71	25.02 ± 19.02	0.339
SGOT (IU/L)		25.44 ± 12.98		25.10 ± 12.59	28.77 ± 15.98	0.010
Uric acid (mg/dL)		5.65 ± 1.42		5.60 ± 1.38	6.19 ± 1.65	< 0.001

Data presented as mean ± standard deviation for continuous variables or n (%) for categorical variables.

*BMI* body mass index, *WC* waist circumference, *WHR* Waist-to-hip ratio, *WHtR* waist-to-height ratio, *FAT* body fat, *SBP* systolic blood pressure, *DBP* diastolic blood pressure, *PP* pulse pressure, *MAP* mean arterial pressure, *TG* triglycerides, *HDL-C* high density lipoprotein-cholesterol, *LDL-C* low density lipoprotein-cholesterol, *FPG* fasting plasma glucose, *BUN* blood urea nitrogen, *eGFR* estimated glomerular filtration rate, *UACR* urine albumin-to-creatinine ratio, *baPWV* brachial-ankle pulse wave velocity, *ABI* ankle-brachial index, *SGPT* serum glutamic-pyruvic transaminase, *SGOT* serum glutamic-oxalocetic transaminase.

^a^There were 25 missing values in leisure activity group; 8 missing values in hypertension, 22 missing values in hyperlipidemia, 4 missing values in diabetes, and 2 missing values in gout.

**Figure 1:**
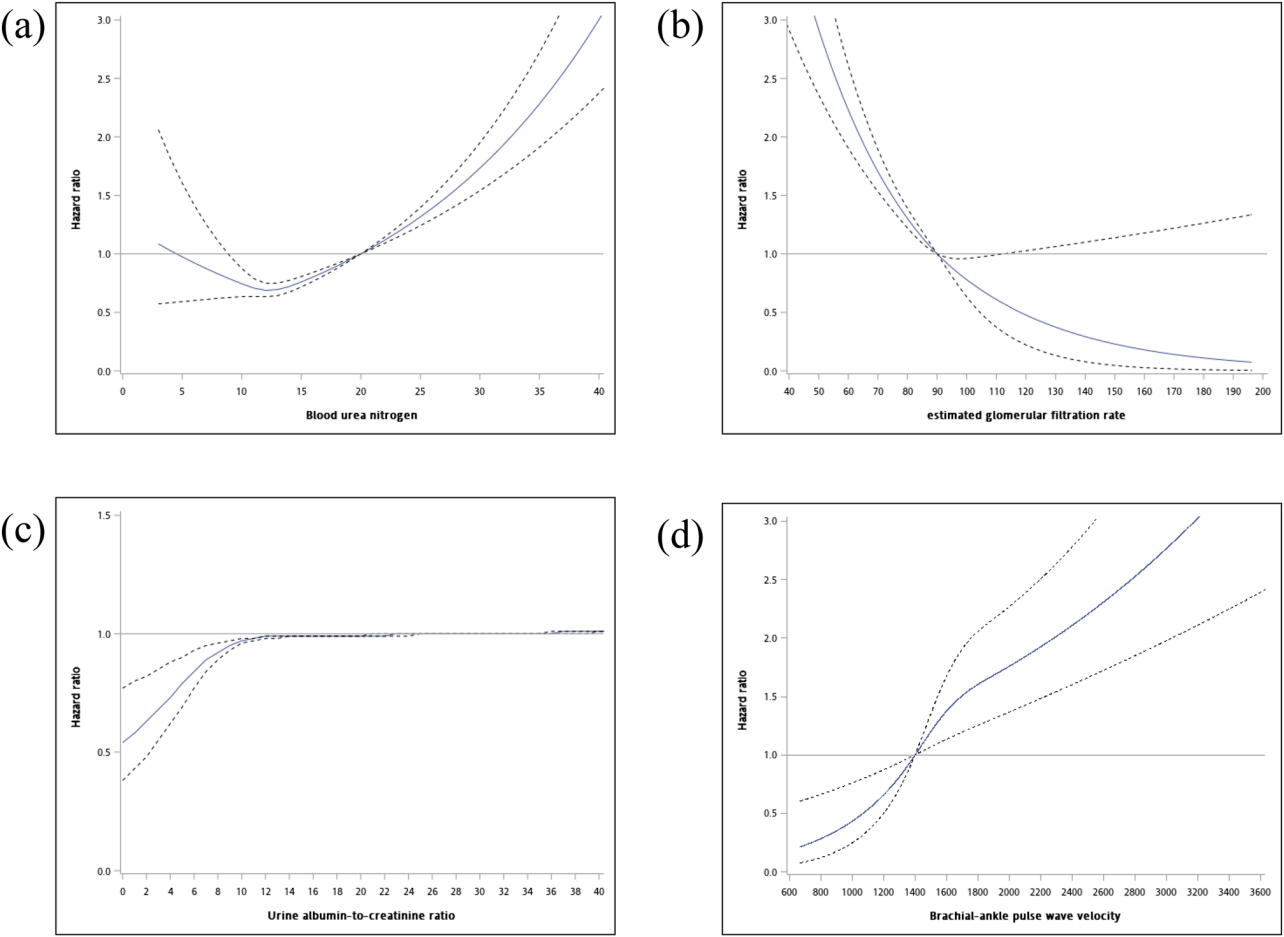
Restricted cubic spline model displaying the hazard ratios for all-cause mortality by (**a**) BUN, (**b**) eGFR, (**c**) urine ACR, and (**d**) baPWV.

**Figure 2:**
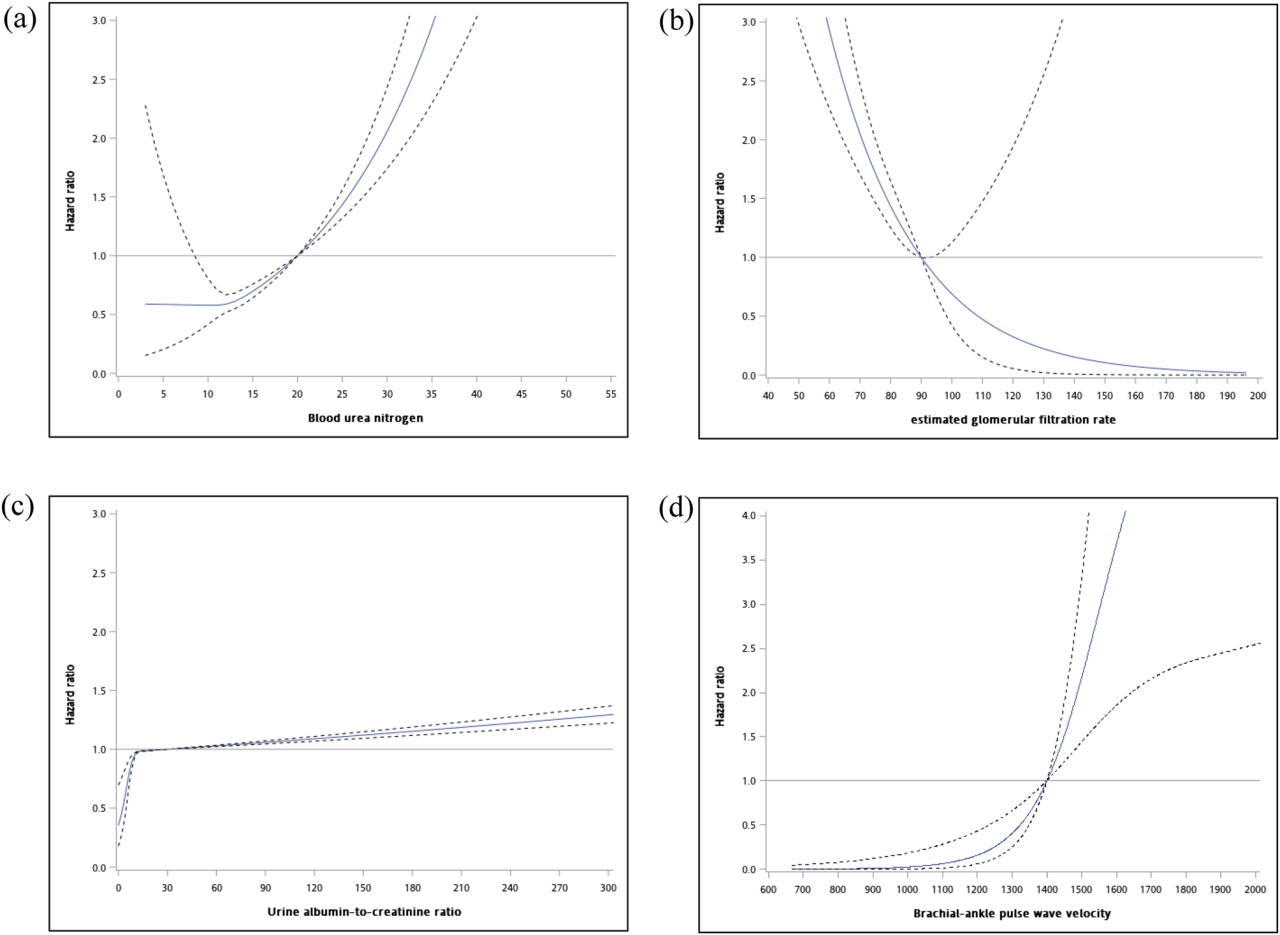
Restricted cubic spline model displaying the hazard ratios for expanded cardiovascular disease mortality by (**a**) BUN, (**b**) eGFR, (**c**) urine ACR, and (**d**) baPWV.

The cut-off points of renal-related variables were then determined. The clinical cut-off points of the laboratory examinations were as follows: eGFR ≥ 90 mL/min/1.73 m^2^ as determined by the criteria of the 2012 Kidney Disease: Improving Global Outcomes (KDIGO) Clinical Practice Guideline^27^, UACR < 30 mg/g as determined by the criteria of the 2012 KDIGO Clinical Practice Guideline^27^, and BUN < 20 mg/dL^28^, all of which were treated as the normal range. The clinical cut-off point of baPWV < 1400 cm/s was treated as the normal range^29^. For the modified cut-off points of renal-related variables, the subgroup with the least risk was treated as the reference group, and the factors that were associated with all-cause mortality were as follows: BUN was < 20 mg/dL, eGFR ≥ 90 mL/min/1.73 m^2^, UACR < 30 mg/g, and baPWV < 1400 cm/s; and for expanded CVD mortality were as follows: BUN was < 20 mg/dL, eGFR ≥ 90 mL/min/1.73 m^2^, UACR < 25 mg/g, and baPWV < 1400 cm/s (Figs. 1, 2 the solid lines represent HRs, and the dashed lines represent 95% CIs from restricted cubic splines analysis in Cox proportional hazards models). After comparing the AIC values of the two models (Supplementary Table S1), BUN, eGFR, and baPWV used the clinical cut-off points for both all-cause and CVD-expanded mortality, whereas UACR used cut-off points for all-cause and modified cut-off points for expanded CVD mortality.

The final multivariable Cox proportional hazard model for all-cause mortality contained renal-related variables along with the significant variables of HDL-C, gender, age group, type of cohort, heart disease, cerebrovascular disease, cancer, smoking habits, educational level, marital status, hours of sleep, pulse, LDL-C, platelet, and hematocrit. Table 2 shows the incidence density rates, HRs, and 95% CIs of renal-related variables with the main effects without adjustment, with full adjustment (model 1) and with full adjustment and baPWV (model 2) for all-cause and expanded CVD mortality. For all-cause mortality, the adjusted HRs in model 1 were similar to those in model 2, indicating that the confounding effects of baPWV were trivial. The multivariable-adjusted HR was 1.55 (95% CI 1.22–1.96) in model 1 and 1.53 (95% CI 1.21–1.94) in model 2 for BUN (≥ 20 mg/dL vs. < 20 mg/dL), 1.62 (95% CI 1.19–2.22) in model 1 and 1.57 (95% CI 1.15–2.14) in model 2 for eGFR (< 90 mL/min/1.73 m^2^ vs. ≥ 90 mL/min/1.73 m^2^), 1.55 (95% CI 1.25–1.93) in model 1 and 1.55 (95% CI 1.25–1.92) in model 2 for UACR (≥ 30 mg/g vs. < 30 mg/g), and 1.75 (95% CI 1.14–2.67) in model 2 for baPWV (≥ 1400 cm/s vs. < 1400 cm/s). For the expanded CVD mortality, the adjusted HRs in model 2 were slightly attenuated compared with those in model 1, indicating that baPWV had a slight confounding effect. The multivariable-adjusted HR of significant variables was 1.91 (95% CI 1.32–2.76) in model 1 and 1.89 (95% CI 1.30–2.73) in model 2 for BUN (≥ 20 mg/dL vs. < 20 mg/dL), 2.67 (95% CI 1.31–5.43) in model 1 and 2.28 (95% CI 1.13–4.57) in model 2 for eGFR (< 90 mL/min/1.73 m^2^ vs. ≥ 90 mL/min/1.73 m^2^), 2.22 (95% CI 1.58–3.11) in model 1 and 2.13 (95% CI 1.52–2.99) in model 2 for UACR (≥ 25 mg/g vs. < 25 mg/g), and 15.73 (95% CI 2.14–115.61) in model 2 for baPWV (≥ 1400 cm/s vs. < 1400 cm/s). The HR of expanded CVD mortality for ACR by using the clinical cut-off point was 2.07 (1.47–2.92).

**Table 2:** Hazard ratios of renal-related variables for all-cause and expanded cardiovascular disease mortality from Cox proportional hazard models.

Key variables of interest	Subjects (n = 4883)		Death (n = 456)		Person-years			Incidence density (1000 person-year)	Hazard ratio (95% confidence intervals)				
									Unadjusted		Model1		Model2^a^
**All-cause mortality**													
**BUN (mg/dL)**													
< 20	4543		358		37,715.12			9.49	1.00		1.00		1.00
≥ 20	340		98		2605.22			37.62	3.98 (3.18–4.97)***		1.55 (1.22–1.96)***^,a^		1.53 (1.21–1.94)***^,a^
**eGFR (mL/min/1.73 m**^**2**^**)**													
≥ 90	2480		62		19,723.27			3.14	1.00		1.00		1.00
< 90	2403		394		20,597.07			19.13	5.88 (4.49–7.68)***		1.62 (1.19–2.22)**^,a^		1.57 (1.15–2.14)**^,a^
**UACR (mg/g)**													
< 30	4253		308		35,332.38			8.72	1.00		1.00		1.00
≥ 30	630		148		4987.96			29.67	3.42 (2.81–4.16)***		1.55 (1.25–1.93)***^,a^		1.55 (1.25–1.92)***^,a^
**baPWV (cm/s)**													
< 1400	1758		31		14,211.92			2.18	1.00		NA		1.00
≥ 1400	3125		425		26,108.42			16.28	7.28 (5.05–10.5)***		NA		1.75 (1.14–2.67)*^,a^
**Expanded cardiovascular disease mortality**													
**BUN (mg/dL)**													
< 20	4543		102		37,715.12			2.70	1.00		1.00		1.00
≥ 20	340		44		2605.22			16.89	6.29 (4.42–8.96)***		1.91 (1.32–2.76)***^,b^		1.89 (1.30–2.73)***^,b^
**eGFR (mL/min/1.73 m**^**2**^**)**													
≥ 90	2480		10		19,723.27			0.51	1.00		1.00		1.00
< 90	2403		136		20,597.07			6.60	12.6 (6.62–23.9)***		2.67 (1.31–5.43)**^,b^		2.28 (1.13–4.57)*^,b^
**UACR (mg/g)**													
< 25	4146		80		34,451.83			2.32	1.00		1.00		1.00
≥ 25	737		66		5868.51			11.25	4.87 (3.51–6.74)***		2.22 (1.58–3.11)***^,b^		2.13 (1.52–2.99)***^,b^
**baPWV (cm/s)**													
< 1400	1758		1		14,211.92			0.07	1.00		NA		1.00
≥ 1400	3125		145		26,108.42			5.55	77.1 (10.8–550)***		NA		15.73 (2.14–115.61)**^,b^

*NA* Not applicable, *M* man, *W* women, *SBP* systolic blood pressure, *BMI* body mass index, *HDL-C* high density lipoprotein-cholesterol, *BUN* blood urea nitrogen, *eGFR* estimated glomerular filtration rate, *UACR* urine albumin-to-creatinine ratio, *baPWV* brachial-ankle pulse wave velocity.

*p < 0.05; **p < 0.01; ***p < 0.001.

^a^Additionally adjusted for gender, age group, type of cohort, heart disease, cerebrovascular disease, cancer, smoking habits, education level, marital status, sleep hours, low density lipoprotein-cholesterol, SBP, BMI, HDL-C, and MAP.

^b^Adjusted for gender, age group, type of cohort, heart disease, cerebrovascular disease, alcohol consumption, sleep hours, and serum glutamic-pyruvic transaminase.

Table 3 shows the adjusted HRs and 95% CIs of significant variables for all-cause and expanded CVD mortality obtained via the sensitivity analysis. Subjects who died in the first year of the follow-up period were excluded to rule out the potential bias of reverse causality. The multivariable-adjusted HR of renal-related variables remained similar; for all-cause mortality, it was 1.38 (95% CI 1.07–1.77) for BUN (≥ 20 mg/dL vs. < 20 mg/dL), 1.61 (95% CI 1.16–2.22) for eGFR (< 90 mL/min/1.73 m^2^ vs. ≥ 90 mL/min/1.73 m^2^), 1.52 (95% CI 1.24–1.86) for UACR (≥ 25 mg/g vs. < 25 mg/g), and 1.62 (95% CI 1.05–2.51) for baPWV (≥ 1400 cm/s vs. < 1400 cm/s); for expanded CVD mortality, it was 2.22 (95% CI 1.06–4.65) for eGFR (< 90 mL/min/1.73 m^2^ vs. ≥ 90 mL/min/1.73 m^2^), 1.86 (95% CI 1.31–2.64) for UACR (≥ 25 mg/g vs. < 25 mg/g), and 14.87 (95% CI 2.02–109.66) for baPWV (≥ 1400 cm/s vs. < 1400 cm/s). The stratified analyses according to the ankle-brachial pressure index (ABI) status (< 0.9 and ≥ 0.9) were shown in Supplementary Table S2. Because the study subjects were sampled in the community, only 64 persons with ABI < 0.9. Thus, no analysis was performed for those with ABI < 0.9. Among those persons with ABI ≥ 0.9, the HRs of BUN, eGFR, UACR, and baPWV remain similar for both all-cause mortality and expanded cardiovascular disease mortality.

**Table 3:** Hazard ratios of key variables of interest for all-cause mortality from Cox proportional hazard model after excluding subjects died in the first year.

Key variables of interest	Subjects (n = 4858)	Death (n = 431)	Person-years	Incidence density (1000 person-year)	Adjusted hazard ratio (95% confidence intervals)^a^
**All-cause mortality**					
**BUN (mg/dL)**					
< 20	4527	342	37,704.87	9.07	1.00
≥ 20	331	89	2597.81	34.26	1.43 (1.11–1.83)**^,a^
**eGFR (mL/min/1.73 m**^**2**^**)**					
≥ 90	2477	59	19,721.61	2.99	1.00
< 90	2381	372	20,581.07	18.07	1.61 (1.16–2.22)**^,a^
**UACR (mg/g)**					
< 30	4240	295	35,323.96	8.35	1.00
≥ 30	618	136	4978.72	27.32	1.52 (1.22–1.90)***^,a^
**baPWV (cm/s)**					
< 1400	1758	31	14,211.92	2.18	1.00
≥ 1400	3100	400	26,090.76	15.33	1.58 (1.02–2.43)*^,a^
**Expanded cardiovascular disease mortality**					
**BUN (mg/dL)**					
< 20	4527	98	37,704.87	2.60	1.00
≥ 20	331	39	2597.81	15.01	1.75 (1.19–2.58)**^,b^
**eGFR (mL/min/1.73 m**^**2**^**)**					
> 90	2477	9	19,721.61	0.46	1.00
≤ 90	2381	128	20,581.07	6.22	2.43 (1.17–5.06)*^,b^
**UACR (mg/g)**					
< 25	4134	77	34,443.99	2.24	1.00
≥ 25	724	60	5858.69	10.24	2.03 (1.43–2.88)***^,b^
**baPWV (cm/s)**					
< 1400	1758	1	14,211.92	0.07	1.00
≥ 1400	3100	136	26,090.76	5.21	14.69 (1.99–108.25)**^,b^

*HDL-C* high density lipoprotein-cholesterol, *BMI* body mass index, *MAP* mean arterial pressure, *BUN* blood urea nitrogen, *eGFR* estimated glomerular filtration rate, *UACR* urine albumin-to-creatinine ratio, *baPWV* brachial-ankle pulse wave velocity.

*p < 0.05; **p < 0.01; ***p < 0.001.

^a^Additionally adjusted for gender, age group, type of cohort, heart disease, cerebrovascular disease, cancer, smoking habits, education level, marital status, sleep hours, low density lipoprotein-cholesterol, SBP, BMI, HDL-C, and MAP.

^b^Adjusted for gender, age group, type of cohort, heart disease, cerebrovascular disease, alcohol consumption, sleep hours, and serum glutamic-pyruvic transaminase.

## Discussion

In this prospective community-based cohort study, we estimated HRs and 95% CIs for the associations between renal-related biomarkers and arterial stiffness markers with all-cause mortality and expanded CVD mortality in the general population of Taichung City, Taiwan. After adjusting for covariates, the participants with higher levels of BUN, UACR, and baPWV and with a lower level of eGFR had a significantly higher risk of all-cause mortality. After conducting the sensitivity analysis and excluding the participants who died in the first year, the results remained similar. With regard to expanded CVD mortality, the participants with higher levels of BUN, UACR, and baPWV and a lower eGFR had a significantly higher risk of expanded CVD mortality after adjusting for covariates. The results of sensitivity analysis demonstrated that the present findings were robust.

In the findings with full adjustment, the adjusted HRs of all-cause mortality indicated that baPWV was a significant predictor, a result consistent with that of a prior study^18^. In the causal pathway for CVD, baPWV, as an indicator of arterial stiffness, is the consequence of renal-related and metabolic syndrome-related disorders. baPWV is a significant predictor, and the effects of SBP are accounted for by baPWV. Therefore, the results implied that abnormalities in SBP lead to an increase in mortality because of the association between mortality risk and baPWV. SBP is an important risk factor for early-stage screening to prevent the development of atherosclerosis and minimize mortality risks. The results of this study did not find associations between metabolic syndrome-related indicators of blood pressure and all-cause mortality or expanded CVD mortality probably because of the limited sample size.

Previous cohort studies of patients with CKD or heart failure that explored renal-related biomarkers demonstrated that high levels of BUN are associated with increased risks of all-cause mortality^7–9^. Population-based or occupational cohort studies found that high levels of UACR are associated with increased risks of all-cause mortality and CVD mortality^13–15^. Two other population-based cohort studies reported that low levels of eGFR are associated with increased risks of all-cause mortality and CVD mortality^10,12^. The present study of renal-related biomarkers had similar findings with previous studies^7–10,12–15^. However, these studies considered BUN alone^7,8^, UACR alone^14,15^, eGFR alone^10–12^, both eGFR and UACR^13^, or both eGFR and BUN^9^. None of them considered these three renal-related variables simultaneously. Thus, the estimated associations between these renal-related variables and mortality may not rule out the confounding effects of other renal-related variables. Although reverse causality is less commonly a problem in cohort studies, reverse causality is possible if the excess mortality was only present for the first year of observation, suggesting that mortality caused the abnormal level of renal markers and arterial stiffness rather than vice versa. To rule out this possibility, we performed a sensitivity analysis that excluded subjects who died in the first year of follow up period. Despite excluding cases of death in the first year of the follow-up period, our findings remained consistent. High levels of BUN were found to be associated with UACR, and low levels of eGFR were associated with increased all-cause mortality and expanded CVD mortality risks.

Previous population-based cohort studies of biomarkers of arterial stiffness showed that high levels of baPWV are associated with increased risks of all-cause and CVD mortality^18,20,21^. However, these studies did not evaluate the possibility of reverse causality^18,20,21^. The present study excluded death cases in the first year of the follow-up period. Nevertheless, our findings remained similar, indicating that the results were robust.

Several plausible explanations may account for the association between renal-related variables and mortality. First, persons with impaired renal function or renal damage may have a higher prevalence of metabolic components, including obesity, dyslipidemia, and, hypertension, as well as complications. In this study, persons with an eGFR of < 90 mL/min/1.73 m^2^ compared with those with an eGFR of ≥ 90 mL/min/1.73 m^2^ had 2.72 times higher incidence of hypertension, 1.27 times higher incidence of dislipidemia, 4.99 times higher incidence of CVD, and 2.97 times higher incidence of coronary artery disease, with a corresponding BUN of 2.36, 1.26, 3.02, and 2.80, respectively, and ACR of 2.18, 1.39, 2.93, and 2.07, respectively. These chronic conditions result in impaired renal function or renal damage, which in turn may exert an independent effect on mortality. Thus, our study found that the three renal-related variables were associated with all-cause and cause-specific mortality. The other possible biological mechanism that may explain the observed associations is that impaired renal function or renal damage results in lower clearance and higher inflammation levels and oxidative stress factors, such as asymmetric dimethylarginine and homocysteine^24^. These factors that were not measured herein may explain the higher risk of mortality for impaired renal function or renal damage. Moreover, impaired renal function or renal damage may deteriorate to hypertension and trigger the rennin–angiotensin system^30^, both of which might also increase mortality. These two potential biological mechanisms may also intensify atherosclerotic burden and raise CVD risks.

Our study was a community-based cohort study conducted in Taichung City, Taiwan. Our study outcomes were adequately assessed by linking them with the national death dataset of Taiwan Ministry of Health and Welfare. We adopted a relatively rigorous approach to reduce missing values and enhance data validity. We simultaneously considered multiple indicators of renal-related biomarkers and arterial stiffness markers into the models to be able to estimate their independent effects by ruling out the confounding effects of other factors. We performed restricted cubic splines analysis in Cox proportional hazards models to explore potential nonlinear associations in addition to clinical cut-off points. We determined which cut-off points had better goodness of fit according to AIC values. All participants in this study came from three cohorts, but anthropometric measurements and laboratory examinations were measured using the same equipment in the same hospital to avoid measurement bias. This study used the cause-specific mortality status to derive expanded CVD mortality, and this composite measure enhanced the power of the present study.

This study has several limitations. First, our findings should be carefully adopted in generalizing them to other areas of Taiwan because all subjects came from a single center. Hence, this study might have had selection bias. Second, all participants came from three cohorts, and this study sample might lack representativeness because the elderly might have been oversampled owing to the inclusion of the TCHS-E study, which specifically included persons aged ≥ 65 years. Thus, the age distribution was not consistent with the age distribution of the population in Taichung. Nevertheless, the combination of these three cohorts in this study increased the sample size and the statistical power. Given that this study was not descriptive, representativeness was not a key issue for consideration. In an analytical cohort study, the key issue for consideration is to have a sample containing an adequate number of subjects with the major predictor characteristics and a sufficient number of subjects with outcome events. The combination of these three cohorts satisfied this consideration. The sample size of the combined cohort was 4883, the power for exploring the associations between renal marker and mortality was over 90% given the assumption of alpha 0.05 and the observed strength of association in the present study. If only one of these three cohorts was used, the maximum power would be 72%. Third, anthropometric measurements and laboratory examination were measured at one time point only in this study. Hence, the effects of these variables over time were not examined. Fourth, our study was an observational study, thus, lacks randomization process. There exists potential bias caused by unknown or residual confounding variables. In this study, we used multivariable analysis to control for the potential confounding effects through adjusting for covariates being reported in literature. Finally, this study lacked precise information on the type of medications the participants take. Thus, this factor was not considered.

In this prospective community-based cohort study in Taiwan, renal-related biomarkers, namely, high levels of BUN and UACR and low levels of eGFR, as well as a marker of arterial stiffness, namely, high levels of baPWV, showed high risks with all-cause and expanded CVD mortality. Our study provides new insights into the conduct of screening tests. UACR < 25 mg/g is suggested as the cut-off point to target populations of high risk to mortality due to CVD in the Chinese general population.

## Methods

### Study population

**Figure 3:**
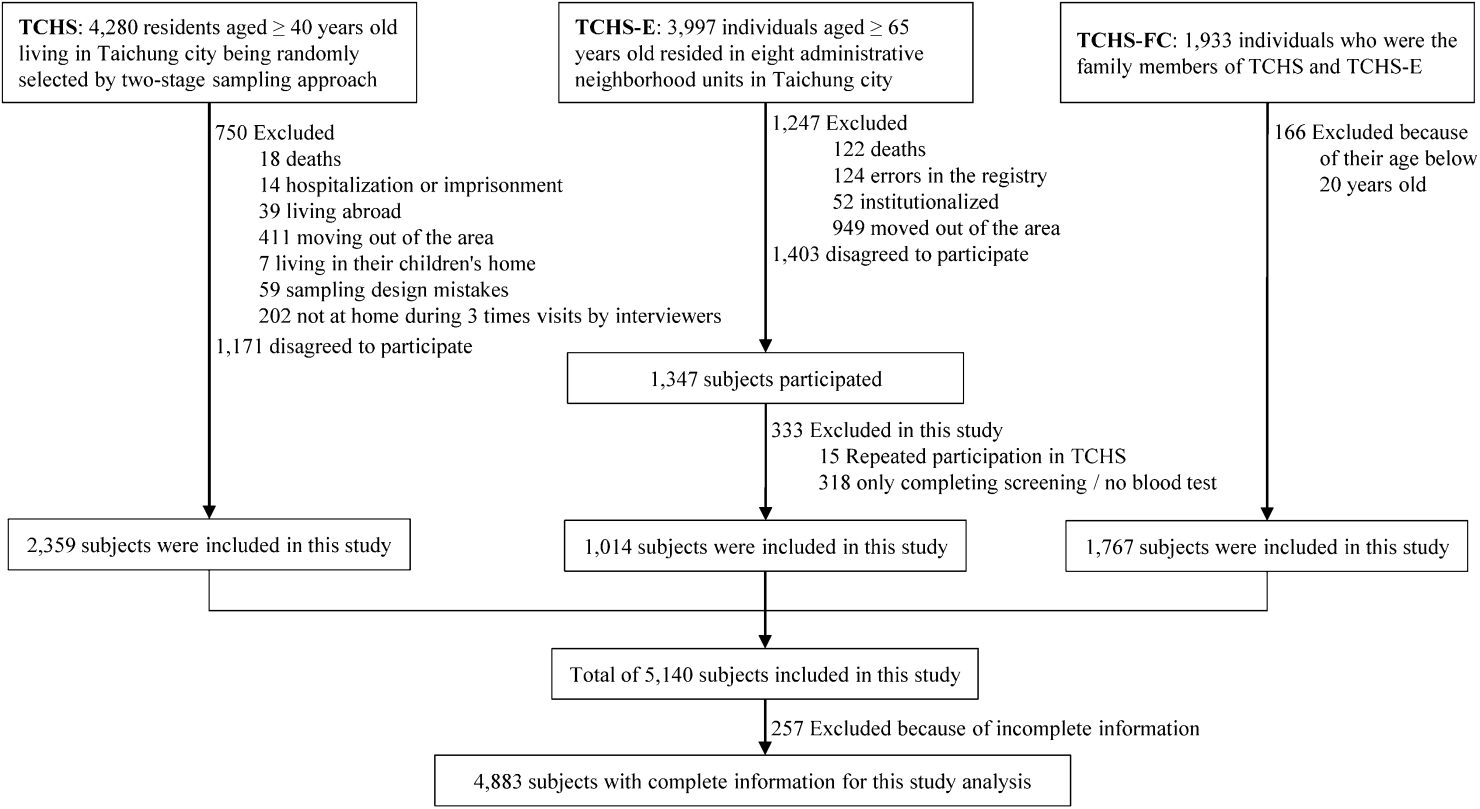
Flowchart of the study’s recruitment. *TCHS* Taichung Community Health Study, *TCHS-E* Taichung Community Health Study-Elderly, *TCHS-FC* Taichung Community Health Study-Family Cohort.

This prospective, community-based cohort study used the database of TCHS, TCHS-E, and TCHS-FC. The recruitment process is outlined in a flowchart given in Fig. 3. The first wave of data collection of these studies was treated as the baseline. The endpoint was set on December 31, 2016. The study population of TCHS included residents of Taichung City, Taiwan aged ≥ 40 years as of October, 2004. Using a two-stage sampling design, TCHS adopted a simple random sampling approach with a sampling rate proportional to size within each stage to draw residents from all eligible individual records of the Households Bureau. A total of 4280 residents were randomly selected from 39 randomly selected administrative neighborhoods (Lis). After excluding 750 ineligible study subjects, a total of 3530 eligible subjects aged ≥ 40 years were finally selected, 2359 of which agreed to participate in October 2004 and formed the baseline population^31^. This population was ultimately included in the present study.

TCHS-E had 3997 residents aged ≥ 65 years who lived in 8 Lis around China Medical University Hospital in North District, Taichung City in June 2009, among which 2750 elderly residents were eligible. A total of 1347 elderly subjects agreed to participate and formed the baseline information of this study^32^. However, 333 subjects were excluded: 15 subjects had repeated participation in TCHS, and 318 subjects had no blood test. Finally, 1014 study subjects were included in this study.

TCHS-FC consisted of participants recruited from the family members (i.e., blood relatives) of those included in TCHS and TCHS-E in October 2010. The family members of the participants were their parents, children, or siblings. A total of 1933 participants aged 12–91 years were recruited from 494 families from 2009 to 2012 and formed the baseline information. In the present study, 166 subjects were excluded because they were aged < 20 years. Finally, 1767 participants were included in this study.

After combining the databases of TCHS, TCHS-E, and TCHS-FC, the total number of subjects included herein was 5140. A total of 4883 subjects with complete information were included in the analysis. This study was approved by the Human Research Committee of China Medical University Hospital (CMUH108-REC1-058) and all methods were performed in accordance with the relevant guidelines and regulations. Written informed consent was obtained from each participant.

### Measurements

#### Anthropometric measurements and laboratory examinations

Blood samples were collected, and anthropometric measurements were conducted during complete physical examination. With the subjects in standing position, barefooted, and wearing light clothing, their weight and height were measured using an autoanthropometer (super-view, HW-666). With the participants in standing position, waist circumference (WC) was measured (to the nearest 1 mm) in the middle between the superior iliac spine and the margin of the rib, whereas hip circumference (HIP) was measured at the maximum point of the buttock point around the pelvis. Blood pressure indicators, including systolic blood pressure (SBP), diastolic blood pressure (DBP) and pulse, were measured using an electronic device (COLIN, VP-1000, Japan). With the subjects lying face upward, the biomarkers of arterial stiffness, namely, brachial-ankle pulse wave velocity (baPWV) and ABI, were measured noninvasively by using a VP-1000 automated PWV/ABI analyzer (PWV/ABI; Colin Co. Ltd., Komaki, Japan) attached to the four limbs. Blood flow was evaluated by estimating the ratio of blood pressure in the legs to blood pressure in the arms by using an ABI analyzer.

After 12 h of fasting, the subjects’ blood was drawn from the anterior elbow vein with minimal trauma in the morning and sent to the Clinical Laboratory Department of China Medical University Hospital for analysis within 4 h of collection. Several biochemical markers, such as hemoglobin, serum glutamic-pyruvic transaminase (SGPT), serum glutamic-oxalocetic transaminase (SGOT), fasting plasma glucose (FPG), creatinine, uric acid, BUN, total cholesterol, TG, HDL-C, LDL-C, and urine albumin, were analyzed using a biochemical autoanalyzer (Beckman Coulter Synchron system, Lx-20, Fullerton, CA, USA). Urinary creatinine (Jaffe’s kinetic method) and albumin (colorimetyl bromcresol purple) were measured using an autoanalyzer. The precision measurement of inter-assay coefficients of variations for both creatinine and albumin concentrations was < 3.0%.

#### Sociodemographic factors, lifestyle behavioral factors, and health status

Data on sociodemographic factors, including age, gender, educational attainment, and marital status, were collected via structured questionnaires administered by interviewers. A person aged ≥ 65 years was defined as elderly. The level of education was classified into five categories: below elementary school, junior high school, high school (including senior high school and vocational high school), junior college, and postbaccalaureate. Marital status was classified into five categories: single, married, widowed, divorced, and separated. A “single” status denoted that the subject never married, whereas a “married” status included married and remarried. Household income was classified into three categories: NT$40,000 or less, NT$40,001–100,000, and > NT$100,001. Information on income was not obtained in the TCHS-E study.

Data on lifestyle behaviors, including smoking habits, alcohol consumption, hours spent sleeping, hours spent sitting, and level of leisure activity, were collected via structured questionnaires administered by interviewers. Smoking habits and alcohol consumption were classified into three groups: never, previously, and presently. The number of hours spent sleeping and sitting was determined by asking the subjects. Level of leisure activity was measured by 26 items, including aerobic sports, ball games, dancing, martial arts, and others, along with the average time spent on each activity and the number of times every week the subjects devoted to them during the past year. Metabolic equivalents (METs, MET hour/week) was calculated using the formula MET of an activity × average duration spent on this particular activity (h) × total number of times every week the subjects engaged in this activity. Leisure activity METs were categorized into two groups, namely, regular activity and no regular activity; subjects with a value of 0 METs was defined as having no regular activity.

Data on medical history were collected via structured questionnaires administered by interviewers. The information on medical history consisted of heart disease, cerebrovascular disease, hyperlipidemia, diabetes, gout, and cancer. A detailed information on the data management for medical history of hyperlipidemia and heart disease is shown in Supplementary Appendix.

#### Renal-related biomarkers, metabolic syndrome-related markers, and arterial stiffness markers

Data on renal-related biomarkers, including renal function and kidney injury indicators, were measured at the Clinical Laboratory Department of China Medical University Hospital. Renal function indicators included BUN and eGFR. Glomerular filtration rate (GFR) is a measure of renal function. GFR measures the level of creatinine (mg/dL) in serum and is calculated to determine the status of kidney function. This study adopted the following formula: eGFR = 141 × minimum (serum creatinine/k, 1)^α^ × maximum (serum creatinine/k, 1)^−1.209^ × 0.993^age^ × (1.018 if female) × (1.159 if black), where *k* = 0.7, *α* =  − 0.329 if female; and *k* = 0.9, *α* =  − 0.411 if male^33^. Kidney injury indicator included UACR. Albuminuria is a marker of increased urinary albumin excretion and kidney injury. UACR was used as a surrogate marker of albumin excretion rate and was measured in urine samples collected in the morning.

Data on metabolic syndrome-related markers, including obesity indicators, blood pressure indicators, blood lipid indicators, blood sugar indicators, and inflammation indicators, were measured at the Clinical Laboratory Department of China Medical University Hospital. Obesity indicators consisted of body mass index (BMI), WC, waist-to-hip ratio (WHR), waist-to-height ratio (WHtR), and body fat (FAT). BMI was calculated as weight (kg) divided by height squared (m^2^). WHR was computed by dividing WC by HIP. WHtR was calculated by dividing WC by height. Blood pressure indicators included SBP, DBP, pulse pressure (PP), and mean arterial pressure (MAP). PP was calculated as the difference between systolic blood pressure and diastolic blood pressure. MAP was calculated as (SBP + [2 × DBP]) ÷ 3. Blood lipid indicators included HDL-C, TG, LDL-C, and total cholesterol. FPG was the only blood sugar indicator considered in the present study. The inflammation indicator considered herein was WBC, which reflects the human body’s nonspecific inflammatory response to infection or injury.

Data on biomarkers of arterial stiffness, including baPWV and ABI, were measured at the Clinical Laboratory Department of China Medical University Hospital. The maximum of the left and right values for baPWV or ABI was chosen. A low ABI value indicates a high level of severity of peripheral artery disease. A high baPWV value denotes a high level of severity of arterial stiffness.

#### Mortality

Cause-specific mortality status was based on the causes of national death data of Taiwan Ministry of Health and Welfare that were ascertained between October 1, 2004 and December 31, 2016. The death status of the subjects was ascertained by matching the information from death records with the identification number and date of birth of the subjects. Causes of death were coded using the International Classification of Diseases 9th revision (ICD-9) and 10th revision (ICD-10) to evaluate the subjects’ underlying death cause for expanded CVD (ICD-9: codes 390–459 for death cause of CVD, code 250 for death cause of DM, and codes 580–589 for death cause of CKD; ICD-10: codes I00–I99 for death cause of CVD, codes E10–E14 for death cause of DM, and codes N00–N07, N17–N19, and N25–N27 for death cause of CKD). The expanded cardiovascular diseases mortality was considered because it was a composite measure of cardiovascular-related mortality, i.e. measurements based on multiple items for cause of death that are cardiovascular-related^34^. We assume that persons with diabetes are more likely to die from diabetes, as well as the other cardiovascular diseases. In addition, CKD has been known as a key risk factor for cardiovascular disease (CVD). Thus, the death of cause due to renal diseases has been considered as CVD-related death. Under this condition, the composite measure has the advantage of the increase in statistical power to detect the association of the interest.

### Statistical analysis

For descriptive statistics, continuous variables were presented as mean ± standard deviation, whereas categorical variables were presented as n (%).Generalized linear models with generalized estimating equations were used to compare the characteristics of the participants between who are dead and alive at baseline by considering the dependence among family members.

Variables related to metabolic syndrome, including obesity indicators of BMI, WC, WHR, and WHtR; blood pressure indicators of SBP, DBP, and MAP; blood lipid indicators of TG and HDL-C; blood sugar indicator of FPG; inflammatory indicator of WBC; and arterial stiffness markers of baPWV and ABI; were treated as covariates. The other covariates included gender; age group; type of cohort (dummy variables for TCHS, TCHS-E, and TCHS-FC); educational level; marital status; smoking habits; alcohol consumption; number of hours spent sleeping and sitting; level of leisure activity; medical history of heart disease, cerebrovascular disease, hypertension, hyperlipidemia, diabetes, gout, and cancer; blood lipid indicators of LDL-C and total cholesterol; and other covariates of pulse, SGPT, SGOT, and uric acid. According to Pearson’s correlation coefficient, the correlation coefficient between LDL-C and total cholesterol was 0.87 (p < 0.001), and the correlation coefficient between SGPT and SGOT was 0.87 (p < 0.001), indicating that the possibility of multicollinearity was unlikely.

Stratified Cox proportional hazards models with unadjusted and adjusted covariates were used to estimate hazard ratios (HRs) and 95% confidence intervals (CIs) for all-cause mortality and expanded CVD mortality by considering the dependence among family members. Survival time for the descendants was defined as the period between the dates for baseline and death; for persons with censoring data (i.e., survivors), survival time was defined as the period between the dates for baseline and end of follow up (December 31, 2016). Restricted cubic splines analysis in Cox proportional hazards models was performed to detect the best cut-off points of each continuous key variable of interest. The multivariable model-building procedures were as follows. First, univariate analysis for all covariates was assessed and covariates with p-value < 0.25 were retained^35^. Second, key variables of interest were individually adjusted for the covariates with p-value < 0.25 in the multivariable analysis to examine whether these key variables of interest were statistically significant (p-value < 0.05). Third, the continuous and significant key variables of interest were classified into categorical variables by clinical cut-off points and modified cut-off points as determined by restricted cubic splines analysis to test their assumption of linearity. Fourth, Akaike information criterion (AIC) values of the two models derived in step 3 were compared along with significant covariates to select which model had the better goodness of fit for each key variable of interest. Fifth, all renal-related variables were treated as categorical and covariates identified in step 3 were simultaneously entered into the multivariable analysis. Except for gender, age group, and type of cohort, non-significant key variables of interest and covariates were removed one by one for the variable with the largest p-value being deleted first. This process was repeated until all retained variables were significant. Sixth, biomarkers of arterial stiffness were added into the model as the final model because arterial stiffness is the consequence of renal-related and metabolic syndrome-related diseases in the causal pathway for CVD.

The possibility of any association resulting from reverse causality was minimized by performing a sensitivity analysis that excluded subjects who died in the first year of follow up period. To rule out the possibility of the impact on ABI on the results, a sensitivity analysis was performed for the Cox’s model by stratifying the ABI status (low ABI: < 0.9; and normal/high ABI: ≥ 0.9). All p-values were two-sided tests, and the level of statistical significance was set at p-value < 0.05. All statistical analyses were performed using SAS version 9.4 (SAS Institute Inc., Cary, NC, USA).

## Supplementary Information

Supplementary Tables.
