# Supplementary material for: Renal markers and risks of all cause and cardiovascular mortality from the Taichung community based cohort study
Source: Sci Rep. 2021 Jul 8;11:14143. doi: 10.1038/s41598-021-93627-5 (PMC8266842; doi:10.1038/s41598-021-93627-5)
Supplement: Supplementary file 1 — Supplementary Tables. [file 41598_2021_93627_MOESM1_ESM.pdf]

Supplementary Table 1. Selecting the cut-off point of each renal-related variables of interest based on Akaike information criterion (AIC) values for all-cause mortality and expanded cardiovascular disease mortality

Key variables of interest	Hazard ratio (95% confidence intervals)	P-value	AIC values		Selected cut-off point
			Clinical cut-off point	Modified cut-off point	
All-cause mortality					
BUN	1.05 (1.04-1.06) [a]	<0.001	6767.789	6773.814	Clinical cut-off point
eGFR	0.97 (0.97-0.98) [a]	<0.001	6772.880	6772.880	Clinical cut-off point
UACR	1.00 (1.00-1.00) [a]	<0.001	6767.563	6767.648	Clinical cut-off point
baPWV	1.00 (1.00-1.00) [a]	<0.001	6776.844	6776.844	Clinical cut-off point
Expanded cardiovascular disease mortality					
BUN	1.07 (1.05-1.09) [b]	<0.001	2076.192	2076.192	Clinical cut-off point
eGFR	0.97 (0.96-0.97) [b]	<0.001	2080.485	2080.485	Clinical cut-off point
UACR	1.00 (1.00-1.00) [b]	<0.001	2068.414	2065.854	Modified cut-off point
baPWV	1.00 (1.00-1.00) [b]	<0.001	2070.066	2070.066	Clinical cut-off point

BUN: blood urea nitrogen; eGFR: estimated glomerular filtration rate; UACR: urine albumin-to-creatinine ratio; baPWV: brachial-ankle pulse wave velocity;

a: Additionally adjusted for gender, age group, type of cohort, heart disease, cerebrovascular disease, cancer, smoking habits, education level, marital status, sleep hours, low density lipoprotein-cholesterol, SBP, BMI, HDL-C, and MAP.

b: Adjusted for gender, age group, type of cohort, heart disease, cerebrovascular disease, alcohol consumption, sleep hours, and serum glutamic-pyruvic transaminase.

Supplementary Table S2. Hazard ratios of renal-related variables for all-cause and expanded cardiovascular disease mortality from Cox proportional hazard models

Key variables of interest	Ankle-brachial index[#] HR (95% CI)	
	<0.9 (n=64)	≥0.9 (n=4818)
**All-cause mortality**		
BUN (mg/dL)		
<20	NA	1.00
≥20	NA	1.54 (1.22-1.96)*** [a]
eGFR (mL/min/1.73m^2)		
≥90	NA	1.00
<90	NA	1.54 (1.13-2.12)** [a]
UACR (mg/g)		
<30	NA	1.00
≥30	NA	1.54 (1.24-1.92)*** [a]
baPWV (cm/s)		
<1400	NA	1.00
≥1400	NA	1.88 (1.21-2.94)* [a]
**Expanded cardiovascular disease mortality**		
BUN (mg/dL)		
<20	NA	1.00
≥20	NA	1.92 (1.32-2.79)*** [b]
eGFR (mL/min/1.73m^2)		
≥90	NA	1.00
<90	NA	2.31 (1.15-4.64)* [b]
UACR (mg/g)		
<25	NA	1.00
≥25	NA	2.11 (1.50-2.97)*** [b]
baPWV (cm/s)		
<1400	NA	1.00
≥1400	NA	15.02 (2.03-110.99)** [b]

[#]: One person didn't have ankle-brachial index value.

*: p <0.05; **: p <0.01; ***: p <0.001; NA: Not applicable; M: Man; W: Women; SBP: systolic blood pressure; BMI: body mass index; HDL-C: high density lipoprotein-cholesterol; BUN: blood urea nitrogen; eGFR: estimated glomerular filtration rate; UACR: urine albumin-to-creatinine ratio; baPWV: brachial-ankle pulse wave velocity

a: Additionally adjusted for gender, age group, type of cohort, heart disease, cerebrovascular disease, cancer, smoking habits, education level, marital status, sleep hours, low density lipoprotein-cholesterol, SBP, BMI, HDL-C, and MAP.

b: Adjusted for gender, age group, type of cohort, heart disease, cerebrovascular disease, alcohol consumption, sleep hours, and serum glutamic-pyruvic transaminase.
